# Uncertainties in Ecosystem Service Maps: A Comparison on the European Scale

**DOI:** 10.1371/journal.pone.0109643

**Published:** 2014-10-22

**Authors:** Catharina J. E. Schulp, Benjamin Burkhard, Joachim Maes, Jasper Van Vliet, Peter H. Verburg

**Affiliations:** 1 Faculty of Earth and Life Sciences, VU University Amsterdam, Amsterdam, the Netherlands; 2 Institute for Natural Resource Conservation, Kiel University, Kiel, Germany; 3 European Commission - Joint Research Centre, Institute for Environment and Sustainability, Ispra, Varese, Italy; 4 Leibniz Centre for Agricultural Landscape Research ZALF, Müncheberg, Germany; Temasek Life Sciences Laboratory, Singapore

## Abstract

Safeguarding the benefits that ecosystems provide to society is increasingly included as a target in international policies. To support such policies, ecosystem service maps are made. However, there is little attention for the accuracy of these maps. We made a systematic review and quantitative comparison of ecosystem service maps on the European scale to generate insights in the uncertainty of ecosystem service maps and discuss the possibilities for quantitative validation. Maps of climate regulation and recreation were reasonably similar while large uncertainties among maps of erosion protection and flood regulation were observed. Pollination maps had a moderate similarity. Differences among the maps were caused by differences in indicator definition, level of process understanding, mapping aim, data sources and methodology. Absence of suitable observed data on ecosystem services provisioning hampers independent validation of the maps. Consequently, there are, so far, no accurate measures for ecosystem service map quality. Policy makers and other users need to be cautious when applying ecosystem service maps for decision-making. The results illustrate the need for better process understanding and data acquisition to advance ecosystem service mapping, modelling and validation.

## Introduction

The benefits that ecosystems provide to society are increasingly acknowledged. Safeguarding these benefits and maintaining, restoring and enhancing ecosystem services (ES) in the future is included as a target in several international policies, such as the 2020 targets of the Convention on Biological Diversity [1], [2]. The European Union (EU) elaborates this target in the European Biodiversity Strategy that aims at maintaining and enhancing ecosystems and their services [3].

Decisions or policies on ES are made based on available information on the status, trends, and spatial distribution of ecosystem service provision. To support such policies, there is, consequently, an increasing demand for accurate maps of the supply and demand of ecosystem services [4], [5]. The European Commission therefore aims to “map and assess the state of ecosystems and their services (…) by 2014” [3] and several attempts to map the supply and demand of ecosystem services have been presented in the literature [5], [6], [7], [8].

To support policy design on ES, indicators are needed to quantify specific targets on maintaining ES and to monitor progress towards these targets. These indicators should pass basic quality criteria. The Impact Assessment Guidelines of the European Commission summarize such criteria in the RACER framework [9]: indicators should be *Relevant* to the objectives to be reached by the target, *Accepted* by staff and stakeholders, *Credible*, unambiguous and easy to interpret, *Easy* to monitor and *Robust* against manipulation. Although not explicitly stated, the “Accepted, Credible and Robust” criteria acknowledge that indicators should provide accurate data.

Due to the lack of direct monitoring data on ES, they are commonly mapped using model-based proxies. Although it is frequently recognized that such maps are crude estimates, there is little discussion on the magnitude of the errors associated with them [10], [11]. Eigenbrod et al. [12] were the first to quantify the magnitude of errors in ES maps for part of the UK and raised concerns about the accuracy of ES maps and about inconsistencies among mapping approaches [12]. Later reviews of ES mapping studies indicated that only a small fraction of the ES mapping studies did address uncertainty in a quantitative way [11], [13]. For example, uncertainties in ES models were addressed by Kozak et al [14] and Lautenbach et al [15] for small case studies. Schulp et al [16] do address uncertainties in a map for pollination services at a EU scale. Nevertheless, there is little knowledge about the influence of the mapping method and input data on the representation of spatial patterns of ecosystem service supply [17]. Most of the mapping studies pay little attention to error propagation [18].

Studies on ecosystem service map validation are lacking [11]. This is attributed to the fact that many ecosystem services cannot be measured directly, resulting in a lack of direct data on the provision of the service. Several ES mapping studies provide a qualitative comparison with independent proxies, e.g. [7], [19], but these are rather indications for the credibility than full validations.

In this paper, we identify uncertainties in continental-scale ecosystem service maps. Based on a systematic comparison of maps for the EU territory for five ecosystem services (climate regulation, flood regulation, erosion protection, pollination, and recreation), we map spatial patterns of agreement and disagreement for the provision of these five services. Secondly, we evaluate the magnitude and sources of uncertainty in current ecosystem service maps. Based on the quantitative evaluation of ES map consistency and validity, we evaluate to what extent the current ES indicators suffice for target setting and evaluation and other forms of policy support and recommend best practices for ES mapping.

## Methods

### Considered studies

**Figure 1:**
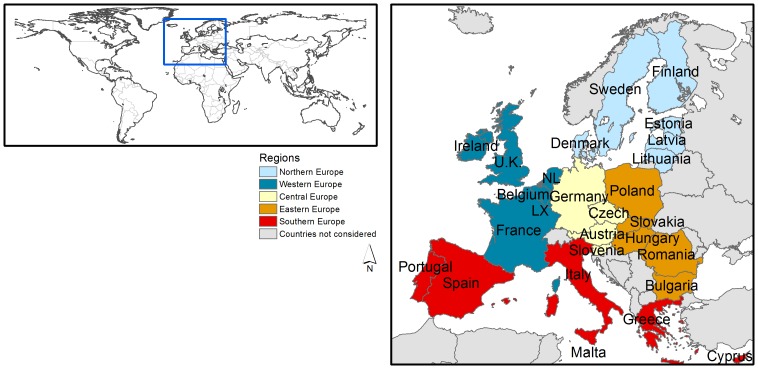
Study area location (left) and regional subdivision and country names as referred to in the results (right). U.K. = United Kingdom, NL = Netherlands, LC = Luxembourg.

To provide insight in the uncertainty in ecosystem service maps, we made an inventory of ES maps that cover the EU extent (Figure 1). For five ES (climate regulation, flood regulation, erosion protection, pollination, and recreation), we identified a wide range of existing maps representing a variety of sources and approaches. For other ES insufficient different maps were available to be included in the analysis.

We analysed uncertainties in ES maps building on four consistent and published sets of ecosystem service maps (Table 1). First, Burkhard et al. [6] map the capacity to provide ES at the European scale using an expert-based classification of land cover data (hereafter referred to as: LC approach). In this approach, an expert evaluation of the level of ES provision for each land cover type into five classes is used to map ES provision. Second, Kienast et al. [7] provide an expert-based map of landscape capacities to provide ES. These maps are based on a similar expert evaluation as the maps of Burkhard et al [6] but include a wide range of environmental variables like relief and landscape types (EV approach). A third set of ES maps originates from a hybrid approach. A hybrid approach uses both process-based models as well as empirical models. This set of ES maps aims to take optimal advantage of available data on land use and environmental indicators [5] (JRC approach). The fourth set of ES maps consists of maps of intermediate complexity. In an intermediate complexity approach, process-based model results are upscaled to a wider extent using empirical relationships with other spatial data [16], [19], [20], [21], [22] (IVM approach).

**Table 1:** Overview of the ecosystem service datasets analysed in this study.

Dataset	Climate regulation	Flood regulation	Pollination	Erosion protection	Recreation
*Datasets included in full analysis*					
LC approach [6]	Capacity of the landscape to provide the service. Based on categorical links between land cover and the service, using CORINE land cover data [23] Categorical, 6 classes ranging from no capacity to very high capacity. 100 m resolution.				
EV approach [7]	Capacity of the landscape to provide the service, expressed as an index based on a set of binary links between environmental variables (including CORINE land cover [23]) and the ecosystem service. Continuous (Dimensionless). NUTS2 resolution.				
JRC approach	Carbon flow, expressed as Net Ecosystem Productivity (NEP). Based on a model based on RS image interpretation [24].	Water quantity regulation: Annually aggregated soil infiltration, derived from a pollutant pathway model. 1 km^2^ resolution [24].	Visitation probability, based on distance decay function from pollinator habitat, multiplied with dependency level of pollinator dependent crops. Based on a crop type map and CORINE land cover [23]. 1 km^2^ resolution [25].	Area based indicator to express the protective function of forests and semi-natural areas based on CORINE land cover [23] in areas with high erosion risk. 1 km^2^ resolution [24]	Capacity of the landscape to provide recreational services. Dimensionless index based on the degree of naturalness, presence of protected areas, distance to coasts, lakes and rivers and bathing water quality. 1 km^2^ resolution [26].
IVM approach	Carbon sequestration, expressed as NEP. Bookkeeping model where detailed flux measurements and simulations are aggregate to country-specific, land use type (based on aggregated CORINE land cover [23]) specific emission factors. 1 km^2^ resolution [20].	Index of flood regulation provision. Based on upscaling of catchment-scale simulations with a process-based hydrological model, to EU scale, using catchment characteristics like land use, topography and soil characteristics. 1 km^2^ resolution [21].	Visitation probability, based on distance decay function from pollinator habitat. Based on CORINE land cover [23] and a map of green linear elements [16].	Protection against erosion by vegetation, based on the Universal Soil Loss Equation and an aggregated version of CORINE land cover [23]. 1 km^2^ resolution [22].	Capacity of the landscape to provide recreational services. Dimensionless index, based on the degree of naturalness; presence of protected areas, presence of coasts, lakes and rivers, presence of High Nature Value farmlands [19].
*Additional maps*					
	Carbon storage: Coupling of global-scale carbon stocks to European-scale land use maps (CORINE land cover [23]) 250 m resolution [24].	Natural hazard reduction: Influence of ecosystem structure on dampening environmental disturbances. Capacity of the landscape to provide the service, following EV approach [7].	Habitat percentage: Area percentage of pollinator habitat. Based on CORINE land cover [23] and a map of green linear elements. 1 km^2^ resolution [16].		
	Net Ecosystem Productivity (NEP) as calculated with the process-based LPJ model for the global carbon cycle. 0.5° resolution [27].		Habitat percentage: Pollinator habitat within a 2 km range of croplands. 1 km^2^ resolution [28].		
*Independent proxy data used for validation*					
Dataset	Global-scale map of NPP, 0.25° resolution [29]. No data for Ireland.	Global-scale map of flood frequency, 1985–2012 [30].	Density of occurrence of wild Apis and Bombus species in northwest Europe [31].	Global-scale map of NPP change over 1980–2003 [32]. No data for Ireland.	Density of inland camping sites [19]
Relation assumed to represent good fit with independent proxy	High NPP coincides with high values of the ecosystem service map	Low frequency coincides with high flood regulation ecosystem service	High pollinator density coincides with high pollination provision	Low NPP loss coincides with high erosion protection	High density coincides with high recreation potential

LC approach: set of ecosystem service maps based on land cover; EV approach: set of ecosystem service maps based on environmental variables. JRC approach: set of data driven ecosystem service maps. IVM approach: set of ecosystem service maps of intermediate complexity.

In addition to these four sets that all contain the same five ES, other studies are available that map one single ES (Table 1). Together, the maps represent the range of complexity of approaches to create ES maps for larger areas.

### Map preparation

Available ES maps strongly differ in representation of the services, units, range of output values and spatial resolution (Table 1). To enable comparison, the maps were made consistent by aggregation to a common spatial resolution and normalizing the ecosystem service indicator values to a common range and unit. Firstly, the categorical LC maps were converted into numerical maps. Following Burkhard et al [33], who indicate that the ES provision categories can be translated into numerical variables using an equal interval classification, we reclassified the ES provision categories of the LC maps into: no ES provision = 0, very low ES provision = 0.2, low = 0.4, moderate = 0.6, high = 0.8, very high = 1. Secondly, all maps were aggregated to NUTS2 regions by calculating the mean value for the ES for each region. The NUTS2 level was chosen as it represents the resolution of the least detailed map. NUTS (the EU Nomenclature of Territorial Units for Statistics) is the standard regional subdivision of the European territory for statistical purposes. NUTS2 regions are the basic regions for the application of regional policies [34]. Thirdly, all maps were normalised using a min-max normalisation to cover the range [0,1] with 0 indicating the lowest value for an ES. The EV maps were computed using a linear combination of explaining variables, and therefore we assumed linearity for the normalization [7].

To summarise maps of individual services, we calculated an ES bundle map for each of the four sets of maps (LC, EV, JRC, IVM). An ES bundle represents an overall level of the provisioning of the five services considered and was calculated as the sum of the five normalised ecosystem service maps. High values thus indicate locations with a relatively high supply or multiple services, while low values indicate the opposite. These bundle maps were included in the comparison because policies aim at protecting the overall level of ecosystem service provision rather than or additional to the provision of individual services [3], [35].

### Map comparison and analysis

Maps for individual ES as well as the bundles were analysed both pair-wise and for all maps together. For the pair-wise comparisons, we use one index that summarizes the relative differences between the maps, the Map Comparison Statistic (MCS) (Equation 1):
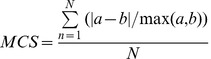
(Equation 1)Where MCS is the Map Comparison Statistic, *a* and *b* are the normalized values of an ecosystem service in a particular NUTS2 region, N is the number of NUTS2 regions considered. MCS values were computed for all available ES maps. This comparison statistic was chosen because it is symmetric (yielding the same result independent of which of the maps is map a or b) and has a defined range (zero for two equal maps; one for two completely contrasting maps) [36]. The MCS thus indicates the average difference between any pair of ES values, expressed as a fraction of the highest value. Two random maps would have an MCS of 0.5 while two opposing maps would have an MCS of 1 and two identical maps would have an MCS of zero.

To analyse the agreement in spatial patterns of ES in the four sets of maps (LC, EV, JRC, IVM), we calculated hotspot and coldspot maps. Hotspots and coldspots are areas providing, respectively, high and low amounts of a particular ES [37], [38] and are defined as areas where the ES supply values fall within the upper or lower quartile of its value distribution. Agreement between the hotspot and coldspot maps of the four mapping approaches was calculated by counting the number of maps that indicated a hotspot or coldspot at a certain NUTS2 region. In addition, we calculated the mean value per NUTS2 region over the four included maps, as well as the coefficient of variation (CV), which is the standard deviation divided by mean. The mean and CV maps were calculated to give a general impression of the spatial patterns of ES supply and their related uncertainty. The mean value over the four included maps gives an indication of the ES supply in each NUTS2 region, while the CV provides information on the relative range of values provided by the different maps, and is therefore an indicator for the patterns of uncertainty in the ES maps.

To support analysis of the sources of uncertainty, ES maps were compared with spatial patterns of land cover. We calculated Pearson's correlations between the percentage of a specific land cover per NUTS2 region and the mean and CV of the mapped supply of each ES. This analysis aimed to explore relations between land cover and ES supply values and the variation in reported values of the ES indicators per land cover type. Although relations between land cover and ES are frequently used for mapping ES, they are largely untested [13] and the exploration therefore was expected to provide useful insights into the variation between ES models for different land cover types.

### Validation

The map comparison methods described in the section “Map comparison and analysis” provide insight in the agreement among the maps and give an indication of the overall level of uncertainty in ES mapping at EU scale. However, it does not provide insight in the deviations from the actual provision of the ES. Therefore, additional to the systematic comparison of ecosystem services maps, the ES maps were compared against independent data that provide a proxy for the ES. As most ES are difficult to measure directly, the independent proxies do not fully match the definition of the ES and not all independent proxies cover the full European Union. However, in all cases association between the spatial patterns of the ES map and the independent proxy can be expected and we interpret such a coincidence association as an indication of the model quality. Table 1 provides an overview of the independent data used for comparison and the assumed relations between the values of the independent map and the ES maps. All independent maps were aggregated to NUTS2 resolution and transformed to ensure the highest values in the independent maps matched the highest values expected from the ES maps. Consistent with Kienast et al. [7], we subdivided the ES map data and the independent data into four quartiles and made a cross-tabulation to calculate the overlap between the independent data and the ES maps. We counted the regions where there was agreement and subdivided this by number of regions where agreement could be expected by chance.

All analyses were performed in R using the packages rgdal [39] and raster [40].

## Results and Interpretation

### Pair-wise map comparisons

Differences among the climate regulation maps are small with MCS values of 0.27 and lower (Table 2). However, when the four maps included in this study are compared to a process-based estimate derived from the LPJ carbon cycle model [27], larger differences are found, with MCS values up to 0.46 for the comparison with the LC map. A map of carbon stocks [41] (Table 1) compares reasonably with all other climate regulation maps; MCS values range between 0.13 (EV map) and 0.24 (LC map). The recreation maps also show relatively small differences among the maps. MCS values for pollination are higher and range up to 0.49 for the comparison between the JRC map and the IVM map. The maps were also compared to two other maps that are an indicator of the available potential pollinator habitat. The map by Serna-Chavez et al. [28] is close to the LC map (MCS: 0.19) but deviates from the JRC map (MCS: 0.44). The habitat map by Schulp et al. [16] is most similar to the JRC map (MCS: 0.19) and deviates most from the IVM map (MCS: 0.38). Flood regulation and erosion protection show high MCS values, indicating that the maps are more different from each other than maps for the other ecosystem services.

**Table 2:** Map comparison statistics of individual ecosystem services and bundles.

Map comparison	Service					
	Climate	Flood regulation	Pollination	Erosion protection	Recreation	Bundle
LC-EV	*0.27*	0.28	0.23	**0.26**	0.28	0.18
LC-JRC	0.18	0.44	0.30	0.26	0.25	0.14
LC-IVM	0.27	**0.17**	0.29	0.45	**0.14**	0.15
EV-JRC	0.20	0.22	0.44	0.40	0.16	0.17
EV-IVM	**0.15**	0.37	**0.20**	0.27	*0.28*	*0.20*
JRC-IVM	0.19	*0.53*	*0.49*	*0.64*	0.26	**0.11**
*Average*	0.21	0.34	0.32	0.38	0.23	0.16

For each service, the *highest* (least similar) and **lowest** (most similar) map comparison statistic are indicated.

For the ecosystem service bundles, the MCS values are lower than for the individual service maps (Table 2). While for the individual services the JRC maps and IVM maps are most deviating, the bundle maps of JRC and IVM are the most similar because differences amongst ecosystem services average out.

### Spatial patterns

**Figure 2:**
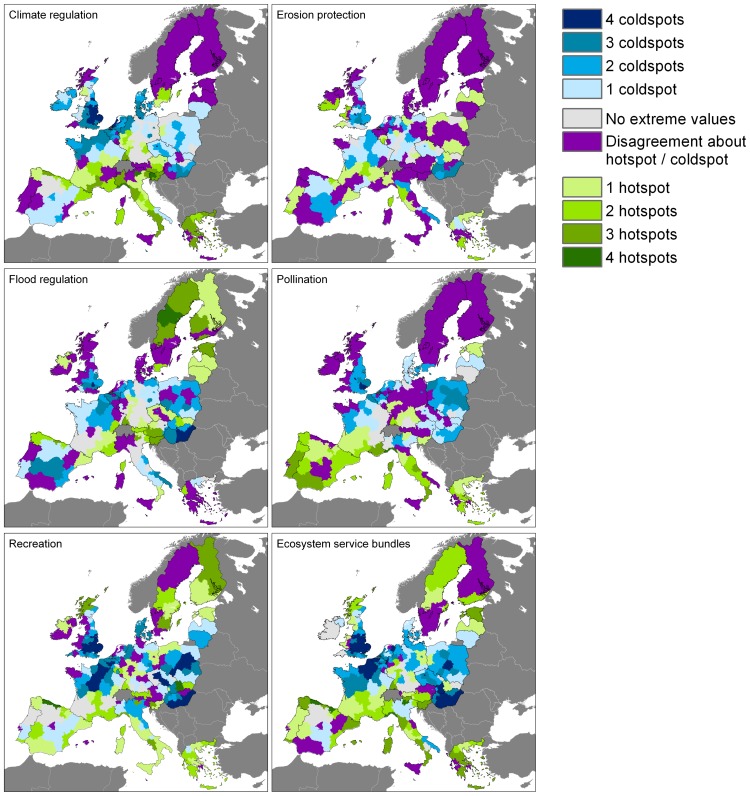
Agreement between maps for each ecosystem service. The maps indicate the number of maps that have a hotspot or coldspot per NUTS2 region. Dark grey areas were not considered.

**Figure 3:**
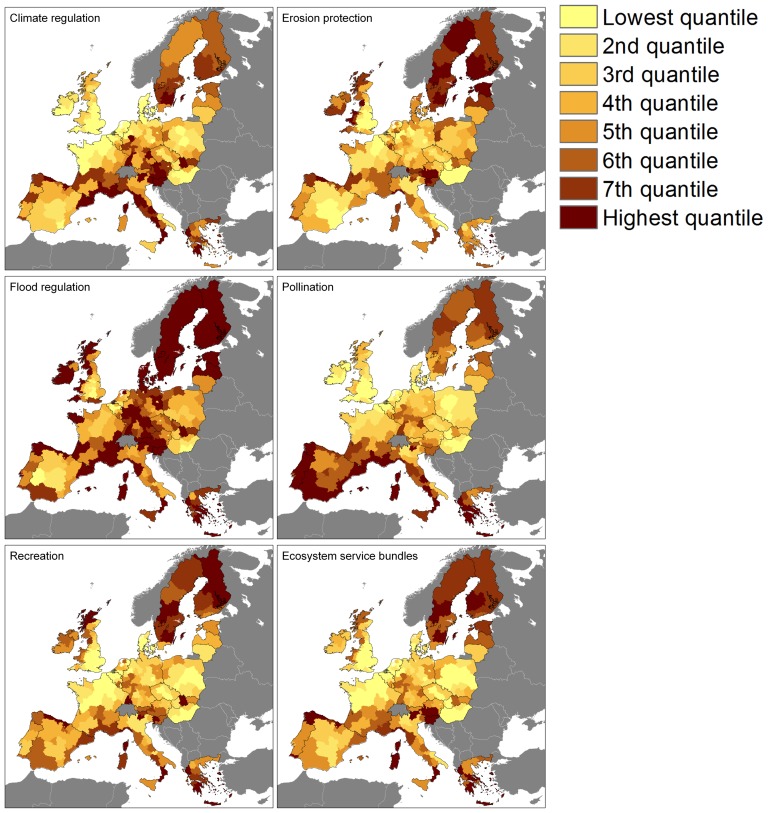
Mean ecosystem service provision per NUTS2 region. Dark grey areas were not considered.

For *climate regulation*, there is agreement on the location of a coldspot in the north-western EU while there is reasonable agreement on hotspots in the central southern region (Figure 2; see Figure 1 for regional subdivision). These coldspots and hotspots can also be seen in the average climate regulation map (Figure 3). High climate regulation capacities are found in Sweden and Finland because of the high percentage forest cover, but here the maps strongly disagree. The provision of climate regulation is strictly defined [42], and is normally quantified based on the rate of carbon sequestration (e.g. in the Common International Classification of Ecosystem Services, CICES (http://cices.eu)). This service is to a large extent provided by natural vegetation. Consequently, the climate regulation maps depend largely on land cover data. All the analysed maps use the same land cover map [23]. The process of carbon sequestration is well-researched [11] and there is consensus on the direction and magnitude of drivers for climate regulation. All maps assume that arable land and urban areas do not provide the service in a relevant amount, and assume that forests and areas that are more natural do. Although the parameterisation of the land cover types differs among the studies, the consistency in input data, well-established process knowledge and strict indicator definition result in the highest level of agreement among the ES assessed here.

*Pollination* maps agree on hotspots in southern Europe and coldspots in western and eastern Europe, while disagreement is seen in central and northern Europe (Figure 2). The areas where the maps disagree have a high average level of pollination provisioning (Figure 3). Indicators for pollination are all based on land cover only, but the maps differ strongly. First, there are differences in definition of the service and in its parameterisation. The JRC approach uses a joint indicator for demand and supply while the indicators used in the LC, EV and IVM approaches focus on supply. The IVM indicator quantifies the probability that a pollinator visits a location while the EV and LC indicators are based on pollinator habitats as proxies. Secondly, pollination depends on landscape configuration and small landscape elements, and with that on the resolution of the input data. The analysed maps are based on different input land cover data and differ in parameterisation of small patches of nature, forest edges, linear landscape elements, and the role of pastures, olive groves and other permanent crops. The EV and LC approach only use the EU-scale CORINE land cover map [23]. The JRC approach supplements this with a map of the density of small patches of nature while the IVM map includes the density of linear landscape elements as an input. As a consequence of the different indicators, parameterisations and input data, high variation among the maps is seen as well as high CV values (Table 3) and a positive correlation of the CV with pasture areas (Table 4).

**Table 3:** Minimum and maximum coefficients of variation for NUTS2 regions between service estimates; low values indicate agreement between the different ES estimates, high values indicate large variation between reported values.

Service	CV			
	Minimum	Location of low values	Maximum	Location of high values
Carbon	0.164	Germany	1.786	Southeast UK, Sweden, Finland
Pollination	0.136	Greece, Spain, Portugal	1.516	Northwest Europe
Erosion protection	0.306	Central Europe and France-Spain border region	1.318	Netherlands, Germany, UK
Flood regulation	0.090	Northern UK, Ireland, Sweden, Finland, Portugal	1.373	Spain, Poland, Hungary
Recreation	0.039	Southern fringes, Germany, Estonia	1.000	Poland, Hungary, UK

**Table 4:** Correlations between area percentages of land cover classes* per NUTS2 region and mean and CV of ecosystem service provision.

		*Urban*	*Pasture*	*Nature*	*Forest*	*Arable*
Carbon	Mean	−0.499	−0.120	0.311	0.777	−0.398
	CV	0.370	0.059	−0.123	−0.439	0.144
Pollination	Mean	−0.525	−0.077	0.438	0.455	−0.307
	CV	0.329	0.340	−0.379	−0.336	0.152
Erosion prevention	Mean	−0.570	0.254	0.304	0.583	−0.428
	CV	0.347	−0.093	−0.466	−0.424	0.548
Flood protection	Mean	−0.533	0.055	0.283	0.609	−0.321
	CV	0.256	−0.084	−0.248	−0.314	0.334
Recreation	Mean	−0.476	0.137	0.481	0.572	−0.570
	CV	−0.013	0.192	−0.292	−0.229	0.363
Bundle	Mean	−0.504	0.082	0.402	0.550	−0.420
	CV	−0.028	0.271	−0.078	0.234	−0.177

*: Urban: all artificial surfaces (CORINE classes 111–142).

Pasture: CORINE class 231. Nature: scrublands, herbaceous vegetation and open spaces (CORINE classes 321–335). Forest: All coniferous/deciduous/mixed forests (CORINE classes 311–313). Arable: All rainfed and irrigated arable land (CORINE classes 211–213).

The *erosion protection* maps show no agreement in regions identified as a hotspot (Figure 2). A few regions show agreement between the coldspots for erosion protection, especially within strongly urbanised regions. This disagreement between the maps for this service is also reflected in the high minimum coefficient of variation (0.31, Table 3). On average, high erosion protection is expected in Sweden and Finland, and in central Europe, due to the high amount of natural vegetation. Low values are found in Hungary, the UK and parts of Spain. In most of the areas with a high average level of erosion protection, the variation among the estimates is large. A variety of indicator definitions is available for this service, that quantify the reduction of soil loss or provide a general indication for the protective effect of natural vegetation [7]. The service depends on many variables, including precipitation, water flow, soil, relief, vegetation and management. This leads to a large variation in input data, as well as model definition and parameterisation, and consequently to a large disagreement between the maps.

The *flood regulation* maps show large differences in their spatial pattern but agree on hotspots in Sweden and Finland and coldspots in Hungary. High mean values are also found in large parts of central Europe while low values dominate in the UK. In considerably large areas with low flood regulation, the maps are in agreement. A variety of indicator definitions is available for this service. Flood regulation can be quantified as the water storage capacity, the reduction of flood danger or damage [43] or as the role of land cover in regulating runoff, discharge, or retention of water [7], [44]. Flood regulation depends on many variables, including precipitation, water flow, soil, relief, vegetation and management. This leads to a large variation in input data, as well as model definition and parameterisation. Finally, flood regulation is a directional service (the service flows from a point of production to a point of use in a specific direction [45], [46], [47]. This is accounted for in the IVM and JRC flood regulation maps but not in the LC and EV maps.

The *recreation* service maps only show small areas of disagreement scattered across Europe. High values are seen along the southern margin of the EU, in the north of the UK, northern Spain and in Sweden and Finland. There is reasonable agreement on the values between the maps (Figure 3) with low coefficients of variation (Table 3). Recreation ES supply is strongly dependent on land cover and the four mapping approaches use the same variables to quantify supply, such as the presence of coasts, protected areas and relief. Also the input datasets are similar in the different approaches, resulting in similar maps.

High overall ES provision is expected in Sweden and Finland and parts of southern Europe while a low provision of the selected services is seen in large urban areas, and in Hungary and the southeast of the UK (Figure 3). The maps do agree on the areas with low values for the ecosystem service bundle. Agreement on areas with high provision of the total bundle is lower (Figure 2).

**Figure 4:**
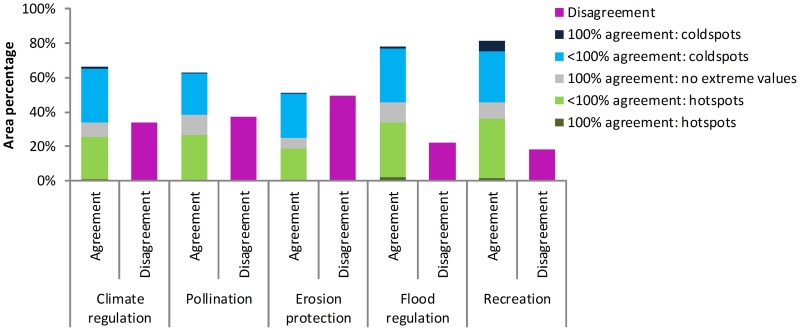
Agreement between the ecosystem service maps. 100% agreement indicates the area where all maps indicate a hotspot, a coldspot or no extreme values, <100% agreement indicates regions where one to three of the maps have a hotspot or coldspot and the other maps do not demonstrate extreme values. Disagreement indicates the regions where at least one map indicates a hotpot and at least one other map indicates a hotspot.

Figure 4 summarizes the area percentage in which the analysed ES maps agree. The erosion protection maps disagree in half of the area considered, meaning that in half of the EU territory some maps expect a hotspot for erosion protection while other maps expect a coldspot at the same location. The recreation maps (partly) agree in >80% of the EU territory. In about 5% of the area, all four analysed maps expect a coldspot for recreation. For all ES, there tends to be more agreement on the locations of coldspots than on the locations of hotspots (Figures 2 and 4). Coldspots for all ES coincide with urban or arable areas. This is supported by more detailed studies that have focussed on the provision of services in arable and urban areas: carbon sequestration [48], pollinator habitat [49], protection against erosion and floods [21] and landscape features for recreational activities [19] are often observed at lower levels in urban or arable areas.

Table 4 summarizes correlations between the mean ES provision values and CVs, and the percentage per region covered by particular land cover types. The provision of all five ES is negatively correlated with regions with a high coverage of urban and arable land, and positively correlated with forests and natural areas. For all individual ES, except recreation, the CVs are positively correlated with the area covered by built-up land, indicating that the maps disagree on ES provision in urban areas. The maps agree on a high level of ES provision in forest areas, indicated by the negative correlations between CVs and forest areas. The positive correlations between CVs and arable land area indicate that the maps disagree on the ES provision in arable land, while for pasture the CV differs per service. In areas with more pasture, the maps disagree more on the provision of pollination and recreational services, while for the other services no relations were found. However, the individual estimates for these areas still show a large variation due to a lack of process understanding and different parameterisations in the models used. The higher agreement of the ES bundle maps is due to averaging out differences between the services.

### Validation

Table 5 summarizes the agreement of the ES maps with independent datasets. For climate regulation, all ES maps compare better with the independent proxy than could be expected by chance. Agreement is mainly seen in parts of northern and southern Europe. For pollination and erosion prevention only one of the ES maps corresponds better with the independent proxy than would be expected by chance. Correspondence of the pollination maps with the independent proxy is seen in mainland western Europe while the erosion maps only show agreement in southern Europe. The flood regulation maps show some agreement with the independent proxy mainly in northern Europe while the recreation maps mainly show some agreement in western Europe.

**Table 5:** Agreement between ecosystem service maps and independent maps.

Map	Climate regulation	Flood regulation	Erosion protection	Pollination	Recreation
LC	1.22	1.33	0.87	0.57	1.18
EV	1.20	1.48	0.92	0.54	1.33
JRC	1.89	0.97	1.14	1.25	0.66
IVM	1.08	1.13	0.95	0.69	0.86

The table shows the ratio between the regions that agree, and the number of regions that would agree by chance.

## Discussion

### Sources of uncertainty

The considerable disagreement among spatial patterns of ecosystem service provision across Europe is an indication of the uncertainties in large-scale ecosystem service assessments. Five sources can contribute to these uncertainties (classified after [18]).

First, the definition of the ecosystem service indicators is not consistent. Different categorisations of ecosystem services are available, the Millennium Ecosystem Assessment [50], the TEEB classification [42] and the CICES classification [51] being the most common. Due to differences in the definition of services in these classification systems, the same service does not necessarily address the same aspects [52], [53].

Secondly, the level of process understanding can cause uncertainty in quantification and mapping. Ecosystem services are supplied by ecosystems to humans through a variety of biophysical and socio-economic processes. Not all these processes are completely understood or quantified [44]. Different levels of understanding and the inherent uncertainty in understanding lead to different quantification methods and different choices regarding the inclusion of determinants.

Third, the aim of mapping influences the selection of the most relevant indicators, the data that are used, and the parameterization of the models.

Fourth, the data sources themselves influence the uncertainty of ES maps. An important data source for all ES maps are land cover data, but also several other biophysical or socio-economic data sources are used. Different ES maps are often based on different data sources to quantify the same input variable. These differences in input data propagate into differences in the resulting ecosystem service maps.

Finally, the methodology for mapping ES is a source of uncertainty. Mapping methods have different levels of complexity, ranging from process-based simulation to expert based value-transfer methods. Different methods result in different ES maps.

The systematic comparison of ES maps indicates that the agreement among ES maps increases when the sources of uncertainty described above are lower. Climate regulation is a clearly defined and well-understood ES which indicators are mostly based on land cover only, resulting in a high agreement. On the other hand, for erosion protection the indicators used for mapping diverge strongly and use a wide variety of input data. As a consequence, the agreement among the maps is lower.

### Validation

By the intercomparison of different ES maps, we provide insight in the spatial patterns of uncertainties and the level of agreement between the maps across Europe. Such intercomparisons of various model outcomes are a common methodology used for different types of global environmental change models, especially for those where independent validation data are scarce [54], [55]. However, model intercomparison does not provide insight in the validity of the models and deviations from the actual ecosystem service provision. Only a comparison of the maps with observed data can help to determine absolute validity levels of the ecosystem service maps.

For the five ecosystem services considered in this paper, available data that can serve as independent proxies to validate the results are collected and compared with the ecosystem service maps. Generally, the studied ES maps are poorly or moderately similar to the independent proxy maps. This does not necessarily provide evidence of the quality of the ES maps assessed here and results have to be interpreted with care. First, the independent proxies are highly different from the ES maps. For none of the ES maps, independent proxies could be identified that exactly match the definition of the service and the indicators used for mapping them. As many ES cannot be measured directly [56], independent proxies that better fit the definitions of the ES indicators were not available. Additionally, there are differences in scale between the maps and independent data. It is not possible to disentangle the relative importance of these two causes from the importance of the map quality upon comparison with independent proxy maps. For a proper validation, independent data covering the variety of conditions throughout the EU would be needed that match the ES definition as described in Table 1. Such data are lacking.

We compared ES maps at the level of administrative units using normalised values of ES provision. Comparing normalized values is potentially less accurate than comparing absolute values. For example, comparison of normalized values would not yield any difference in the case were values for ES provisioning are overestimated by a certain percentage in all locations. However, because the ESs compared in this study were expressed using different units for the same ES, a comparison based on absolute values was not possible. The harmonization of the data to allow comparison has a few other disadvantages as well. Aggregating maps to the resolution of administrative units can lead to a reduction of the spatial variation in ES provision and is likely to result in higher levels of agreement between the maps, especially for ecosystem services showing high spatial variation (e.g. erosion risk, pollination), this can have a large impact.

### Importance for policy making

Robust, reliable and comparable data on ES are important for the advancement of biodiversity objectives and to inform the development and implementation of related policies, on water, climate, agriculture, forest, and regional planning. As elaborated by Maes et al. [51], ES maps at EU scale can support decision-making and implementation in multiple ways. First and foremost, mapping ES is an essential part of the EU Biodiversity Strategy to 2020 and ES maps are used to identify priorities for ecosystem restoration and enable the development of an initiative on no net loss of biodiversity and ES. Importantly, the biodiversity strategy aims to mainstream ES into other policies [41], which also entails the use of ES maps for implementation and targeting of those policies. In the EU, these are notably the common agriculture policy (CAP) and the cohesion policy. The CAP has a profound influence on ecosystems and biodiversity [57] that is recognised in a reform of the policy in 2014, by introducing ecological focus areas aiming at protecting farmland biodiversity and small landscape elements. ES maps will prove to be crucial elements in the spatial identification of those areas where regulating ES support (e.g. pollination, control of pest species, erosion control) and enhance sustainable agricultural production. The cohesion policy, which is essentially responsible for the main share of the EU's investments in the regional economy, is aligning its objectives with goals on sustainable growth. In particular, the conservation and enhancement of natural assets through the development of a green infrastructure network at multiple scales can give rise to socio-economic benefits, which is a priority of cohesion policy. Hence again the need for high quality and consistent spatial information on the levels of services provided by ecosystems which are essential to decision making on future investments using regional EU funds.

## Conclusions

This study showed that a different definition of an ecosystem service or a different mapping approach could lead to strongly different spatial patterns of ecosystem service provision. The systematic intercomparison of four EU-scale maps of different ecosystem services demonstrated that there is an overall agreement among the climate regulation maps and the recreation potential maps. The erosion protection and flood regulation maps differed strongly, the pollination maps showed intermediate variation among the maps. Differences between the maps are caused by differences in the mapping aim, indicator definitions, input data and mapping approaches. The sources of uncertainty differ in their importance for the mapping of different ecosystem services. For services with larger differences in definition and mapping approaches, larger differences between individual maps emerge.

Due to the lack of independent data on ecosystem service provision, ecosystem service maps cannot be properly validated and there are, so far, no appropriate measures for map quality.

The choice of a specific ecosystem service map to support policy will influence the specification of policy targets and implementation priorities. Together with the lack of insight in ecosystem service map quality, varying map compilation and interpretation skills, this indicates that mapmakers and end-users should be cautious when applying ecosystem service maps for decision-making. Mapmakers need to clearly underpin the indicators used, the methods, and related uncertainties. Finally, there is an urgent need for better process understanding and data acquisition for ecosystem service mapping, modelling and validation.
